# Supplementary material for: Older adults with non-communicable chronic conditions and their health care access amid COVID-19 pandemic in Bangladesh: Findings from a cross-sectional study
Source: PLoS One. 2021 Jul 29;16(7):e0255534. doi: 10.1371/journal.pone.0255534 (PMC8320993; doi:10.1371/journal.pone.0255534)
Supplement: S1 File — (DOCX) [file pone.0255534.s002.docx]

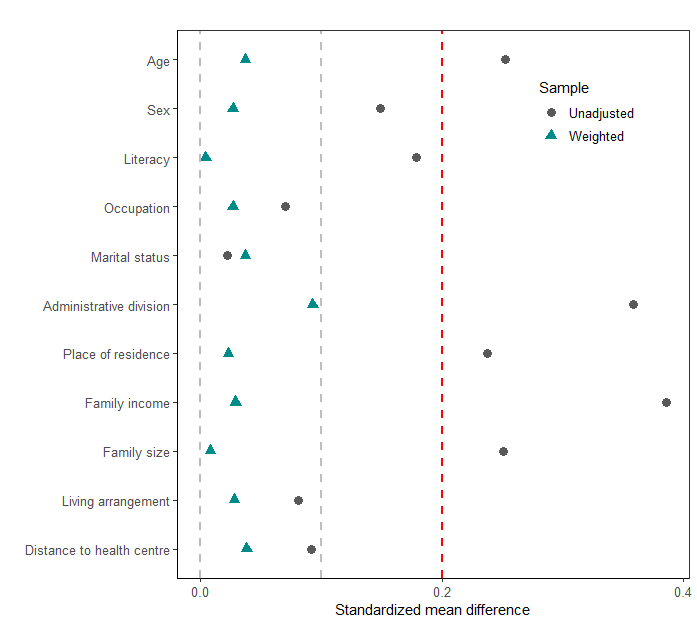


S1 File: Standardized mean differences (SMD) between no non-communicable chronic condition, at least one condition, and multimorbidity in unadjusted and propensity score weighted samples.
