# Supplementary material for: Older adults with non-communicable chronic conditions and their health care access amid COVID-19 pandemic in Bangladesh: Findings from a cross-sectional study
Source: PLoS One. 2021 Jul 29;16(7):e0255534. doi: 10.1371/journal.pone.0255534 (PMC8320993; doi:10.1371/journal.pone.0255534)
Supplement: S2 File — (DOCX) [file pone.0255534.s003.docx]

S2 File: Sensitivity analysis using propensity score analysis for the association between multimorbidity and difficulties receiving medical support among Bangladeshi older adults during COVID-19.

|  | | Experienced difficulties getting medicine | Experienced difficulties receiving routine medical care |
| --- | --- | --- | --- |
|  |  | AOR ^1^ (95% CI) | AOR ^1^ (95% CI) |
| Non-communicable chronic conditions | | |  |
|  | No-condition | Ref. | Ref. |
|  | At least one condition | 2.19 (1.43-3.37) | 3.79 (2.46-5.85) |
|  | Multimorbidity | 4.87 (3.14-7.55) | 6.54 (4.13-10.34) |

AOR: adjusted odds ratio; CI: confidence interval; Ref.: reference category.

^1^ The outcome model was the binary logistic regression on the propensity score weighted data, adjusted for age, sex, marital status, formal schooling, family size, income, administrative division, residence, current occupation, living arrangement, proximity to the nearest health centre.
