# Supplementary material for: Older adults with non-communicable chronic conditions and their health care access amid COVID-19 pandemic in Bangladesh: Findings from a cross-sectional study
Source: PLoS One. 2021 Jul 29;16(7):e0255534. doi: 10.1371/journal.pone.0255534 (PMC8320993; doi:10.1371/journal.pone.0255534)
Supplement: Attachment — Submitted filename: Review response_R1.docx [file pone.0255534.s004.docx]

**Re: "** **Older adults with non-communicable chronic conditions and their health care access amid COVID-19 pandemic in Bangladesh: findings from a cross-sectional study"

Dear Editor,**

We greatly appreciate the valuable comments from the Editor and the reviewers and have modified the enclosed manuscript accordingly. Here, we include our responses to each of the reviewers’ comments.

**Editor**

1. Please ensure that your manuscript meets PLOS ONE's style requirements, including those for file naming. The PLOS ONE style templates can be found at

<https://journals.plos.org/plosone/s/file?id=wjVg/PLOSOne_formatting_sample_main_body.pdf> and

https://journals.plos.org/plosone/s/file?id=ba62/PLOSOne_formatting_sample_title_authors_affiliations.pdf

**Author’s response:** We confirm that the manuscript complies with the PLOS One guidelines

**Reviewer 1**

1. Definition of NCDs is unclear (Table 1). I was concerned about the difference between taking some medications and self-reported. Although both are inherently self-reported, in the case of "Self-reported"(not taking medication or therapy), how did the authors determine if they need to go to hospital or clinic.

**Author’s response:** Thank you very much for bringing this to our notice, and we apologize for the ambiguity. We have revised our statements for clarity by adding the questions that were asked to the participants. Our revised paragraph reads as below on page 7 line 171-179 of the revised manuscript.

*“The data on nine non-communicable chronic conditions (Hypertension, Heart diseases, Stroke, Hypercholesterolemia, Diabetes, Chronic respiratory diseases, Chronic kidney disease, Cancer, and Osteoarthritis) were collected through participants’ self-reports. For each of the conditions, participants were asked two questions: 1) “Has a doctor or other health professional ever told you that you have [the condition]?” and 2) “Are you currently taking any medications for [the condition]?” Participants were classified as having a given condition if they reported yes to either of the two questions. The definitions of the individual non-communicable condition, having at least one condition and multimorbidity are presented in table 1.”*

2. Could you provide the average or median of multimorbidity? I am interested in that data because there is a differnce from a minimum of 2 to a maximum of 9.

**Author’s response:** Thank you. The median of multimorbidity was 2 which have been added in the in the revised manuscript (Result section under sub-heading “Prevalence of at least one condition and multimorbidity”). Please see page 12 line 246-248.

**Reviewer 2**

Authors focused a burning and time demanding topic to discuss in this pandemic. Methodology was clearly discussed, findings were presented appropriately. Please, edit the conclusion section of abstract.

**Author’s response:** Thank you very much for appreciating our paper. We have edited the conclusion of the abstract as suggested.

**Concluding Remarks**

We are extremely appreciative of the Editor’s and reviewers’ time and helpful comments and hope that our revisions have adequately addressed their concerns. We are confident that the revisions have strengthened our manuscript. We look forward to hearing from you with a final decision regarding the acceptance of our manuscript.

Regards,

Authors
